# Optimization of Mechanical Tissue Dissociation Using an Integrated Microfluidic Device for Improved Generation of Single Cells Following Digestion

**DOI:** 10.3389/fbioe.2022.841046

**Published:** 2022-02-08

**Authors:** Marzieh Aliaghaei, Jered B. Haun

**Affiliations:** ^1^ Department of Chemical and Biomolecular Engineering, University of California, Irvine, Irvine, CA, United States; ^2^ Department of Biomedical Engineering, University of California, Irvine, Irvine, CA, United States; ^3^ Department of Materials Science and Engineering, University of California, Irvine, Irvine, CA, United States; ^4^ Center for Advanced Design and Manufacturing of Integrated Microfluidics, University of California, Irvine, Irvine, CA, United States; ^5^ Chao Family Comprehensive Cancer Center, University of California, Irvine, Irvine, CA, United States

**Keywords:** microfluidics, tissue processing, dissociation, single cell analysis, tissue engineering

## Abstract

The dissociation of tissue and cell aggregates into single cells is of high interest for single cell analysis studies, primary cultures, tissue engineering, and regenerative medicine. However, current methods are slow, poorly controlled, variable, and can introduce artifacts. We previously developed a microfluidic device that contains two separate dissociation modules, a branching channel array and nylon mesh filters, which was used as a polishing step after tissue processing with a microfluidic digestion device. Here, we employed the integrated disaggregation and filtration (IDF) device as a standalone method with both cell aggregates and traditionally digested tissue to perform a well-controlled and detailed study into the effect of mechanical forces on dissociation, including modulation of flow rate, device pass number, and even the mechanism. Using a strongly cohesive cell aggregate model, we found that single cell recovery was highest using flow rates exceeding 40 ml/min and multiple passes through the filter module, either with or without the channel module. For minced and digested kidney tissue, recovery of diverse cell types was maximal using multiple passes through the channel module and only a single pass through the filter module. Notably, we found that epithelial cell recovery from the optimized IDF device alone exceeded our previous efforts, and this result was maintained after reducing digestion time to 20 min. However, endothelial cells and leukocytes still required extended digestion time for maximal recover. These findings highlight the significance of parameter optimization to achieve the highest cell yield and viability based on tissue sample size, extracellular matrix content, and strength of cell-cell interactions.

## Introduction

Dissociation of aggregated particulates is a fundamental process in diverse scientific fields including polymer suspensions (Petka et al., 1998), microbeads/nanoparticles (Mafuné et al., 2001), and various cellular constructs in the life sciences (Lindvall and Kokaia, 2006). Currently, the need for efficient disaggregation is particularly strong for tissue and organ samples to help facilitate powerful single cell analysis technologies (Watanabe et al., 2007; Heath et al., 2016). Traditional diagnostic methods provide information about biological traits that have been averaged over an entire population of cells, which masks cell-to-cell variability and the presence of rare cell populations (Tung et al., 2017). This necessitates the analysis of individual cells, which can then be evaluated globally to better understand normal tissue function and diseased states such as cancer (Heath et al., 2016). Towards this goal, single cells must be liberated from tissues efficiently without changing viability or activation state (Nguyen et al., 2018). Cell aggregate dissociation is also needed for regenerative medicine, as current methods can alter stem cell fate and viability (Watanabe et al., 2007; Didar et al., 2013). Therefore, continued development and refinement of rapid and efficient methods for processing tissues and cell aggregates into single cells is a major area of need in the biotechnology and medical arenas.

The traditional method for preparing single cells from tissue includes 1) mincing to reduce tissue size, 2) digesting with enzymes to break down the extracellular matrix and/or cell-cell junctions, 3) mechanically dissociating to release cells, and 4) filtering to remove remaining aggregates. Long term chemical exposure may cause transcriptional and/or proteomic changes, and incomplete dissociation may enrich for certain cell types in the final suspension at the cost of others that may be more challenging to liberate (Mahat et al., 2016; Ayata et al., 2018; Mattei et al., 2020). Importantly, high levels of mechanical stress needed to release cells from deep inside the aggregates can also damage cells, and subsequently reduce yield and/or viability. Hence, there is a critical need to develop and refine methods that will provide well-controlled environmental factors including chemical exposure time and hydrodynamic shear stress level to uniformly release cells from aggregates with minimum damage. Microfluidic systems can facilitate precise manipulation of cell aggregates to achieve high-throughput, cost-effective, and tunable methods (Huang et al., 2004; Yeo et al., 2011; Gubala et al., 2012). However, only a few systems have been developed for dissociation of tissue and cellular aggregates (Wallman et al., 2011; Lin et al., 2013; Ahmed et al., 2014; Qiu et al., 2015; Qiu et al., 2018a; Qiu et al., 2018b; Al-Mofty et al., 2021). Among these, our team has developed three different microfluidic devices that can perform the entire dissociation process workflow including digestion, dissociation, and filtration (Qiu et al., 2015; Qiu et al., 2017; Qiu et al., 2018a; Qiu et al., 2018b). We recently combined all three technologies into a platform and demonstrated improved release of single cells from several tissue types (Lombardo et al., 2021). We also integrated the dissociation and filtration modules into a monolithic device, and since both modules can contribute to breaking down cellular aggregates, we will refer to this as the Integrated Disaggregation and Filtration (IDF) device. Our previous study was focused primarily on optimization of the digestion device, with the IDF device primary used as a final polishing step. Notably, the IDF device was not tested with tissue that was digested in a traditional manner, which could be a format of high interest to some researchers due to operational simplicity and ease of integration into established workflows. Moreover, we believe that the IDF device could serve as an ideal platform for a controlled study into the role of mechanical forces on dissociation of diverse tissue and cell aggregate samples. This is because the IDF device allows for variation of processing parameters, such as flow rate and device pass number, as well as the mode of dissociation via the microchannel array, the nylon mesh membranes, or both in concert. Such testing would result in optimization of mechanical dissociation to maximize cell yield, as well as potentially reduce proteolytic digestion time and allow for parameter modulation to compensate for differences between samples in terms of extracellular matrix (ECM) density, cell-cell adhesion strength, and secondary structures such as vessels and ducts.

In this study, we evaluate the IDF device with samples ranging from cell culture aggregates to minimally digested tissue in an effort to better understand and optimize mechanical dissociation. We first test small, strongly cohesive aggregates produced from the MCF-7 cell line, and show that single cell recovery is maximal at flow rates greater than 40 ml/min. We also find that the filtration module exerts a stronger dissociation effect than the branching channel array, with multiple passes through the filters producing the highest yield. We then employ minced and digested murine kidney, and observe that the primary dissociation mechanism shifts to the branching channel array, with a single filter pass now producing the best results. Under these condition, the IDF device releases as many epithelial cells following minimal digestion (i.e., 20 min) as a full digestion (i.e., 60 min), if device pass number is increased from 10 to 20 passes to compensate. However, this result does not extend to endothelial cells, which appear to have a greater reliance upon enzymatic digestion. This work confirms that the IDF device provides distinct mechanisms for dissociation that depend on aggregate/tissue size, cell-ECM interactions, and cell-cell adhesion. Importantly, the IDF device increases single cell recovery for all samples and cell subtypes, by at least 2-fold, following both short- and long-term digestion periods.

## Materials and Methods

### Device Fabrication

The integrated disaggregation/filtration (IDF) device was fabricated by ALine, Inc. (Rancho Dominguez, CA), as previously described (Lombardo et al., 2021). Briefly, fluidic channels, vias, and openings for fittings were laser machined into 250 µm thick polyethylene terephthalate (PET) layers. Nylon mesh membranes were purchased from Amazon Small Parts (15 and 50 μm pore sizes; Seattle, WA) as large sheets and were laser cut into 8.76 mm diameter circles. Then, the PET layers and nylon mesh membranes were sandwiched between two additional layers of PET, the top containing holes for placement of hose barbs. All layers are aligned and bonded with pressure sensitive adhesive using pressure lamination.

### Cell Culture and Tissue Models

MCF-7 cells were purchased from ATCC (Manassas, VA) and cultured at 37°C and 5% CO_2_ in tissue flasks containing DMEM media with 10% FBS, non-essential amino acids, 1 mM sodium pyruvate, 2 mM L-glutamine, 100 μg/ml streptomycin, 100 U/ml penicillin, and 44 U/L Novolin R insulin (Thermo Fisher, Waltham, MA). Prior to experiments, MCF-7 cell monolayers were briefly treated with trypsin-EDTA to release cells as aggregates and washed with PBS containing 1% BSA (PBS^+^). For tissue dissociation studies, kidneys were harvested from freshly sacrificed C57Bl/6J mice (Jackson Laboratory, Bar Harbor, ME) that were deemed waste from a research study approved by the University of California, Irvine, Institutional Animal Care and Use Committee (courtesy of Dr. Angela G. Fleischman). A scalpel was used to mince the tissue into ∼1 mm^3^ pieces. Then, approximately 10 mg of minced tissue was placed within a conical tube with 300 μl of 0.25% collagenase type I (C9263, Sigma Aldrich, United States). After digesting at 37°C in an incubator under gentle agitation by a rotating mixer for 20–60 min, 700 μl of PBS^+^ was added to deactivate the enzyme. Controls were dissociated using conventional methods comprised of repeated vertexing and pipetting to mechanically disrupt aggregates and filtration with a cell strainer (35 μm) to remove cell debris. All cell suspensions were treated with 100 Units of DNase I (Roche, Indianapolis, IN) for 10 min at 37°C, washed, and resuspended for further analysis.

### Dissociation Studies

Devices were prepared by affixing 1/32 in ID tubing (Nalgene, Rochester, NY) to the hose barbs at both the inlet and outlet. Prior to use, devices were primed with PBS+ and incubated for 15 min to prevent non-specific cell adhesion to the channel walls. Aggregate and tissue models were passed through the microchannel array and/or nylon mesh filter modules of the IDF device under different flow rates (20–60 ml/min) and/or device pass numbers (5–20 passes) by a syringe pump. Finally, devices were flushed with 2 ml PBS^+^ to wash out remaining cells and both device effluents were combined.

### MCF-7 Cell Count and Viability

Cell suspensions from dissociated MCF-7 samples were analyzed for the number of single cells and aggregates using a hemocytometer. Viability was also assessed using Trypan blue stain. The single cell and aggregate counts for each dissociation condition were normalized to the values prior to device processing.

### Flow Cytometry

Kidney cell suspensions were evaluated for epithelial cells, endothelial cells, leukocytes, and red blood cells by flow cytometry, as described (Lombardo et al., 2021). Briefly, cells were stained concurrently with 5 μg/ml anti-mouse CD45-AF488 (clone 30-F11), 7 μg/ml EpCAM-PE (clone G8.8), and 5 μg/ml TER119-AF647 (clone TER-119) monoclonal antibodies (all from BioLegend, San Diego, CA) for 30 min. Samples were then washed twice using PBS + by centrifugation, stained with 3.33 μg/ml of viability dye 7-AAD (BD Biosciences, San Jose, CA) on ice for at least 10 min, and analyzed on a Novocyte 3000 Flow Cytometer (ACEA Biosciences, San Diego, CA). Flow cytometry data was compensated using compensation beads (Invitrogen, Waltham, MA). Gates encompassing the positive and negative subpopulations within each compensation sample were inputted into FlowJo (FlowJo, Ashland, OR) to automatically calculate the compensation matrix. A sequential gating scheme was used to identify live and dead single epithelial cells from leukocytes, red blood cells, non-cellular debris, and cellular aggregates. Signal positivity was determined using appropriate Fluorescence Minus One (FMO) controls. All cell counts were normalized to the mass of tissue that was dissociated.

### Statistics

Data are represented as the mean ± standard error. Error bars represent the standard error from at least three independent experiments. *p*-values were calculated from at least three independent experiments using students *t*-test.

## Results and Discussion

### IDF Device Features

**FIGURE 1:**
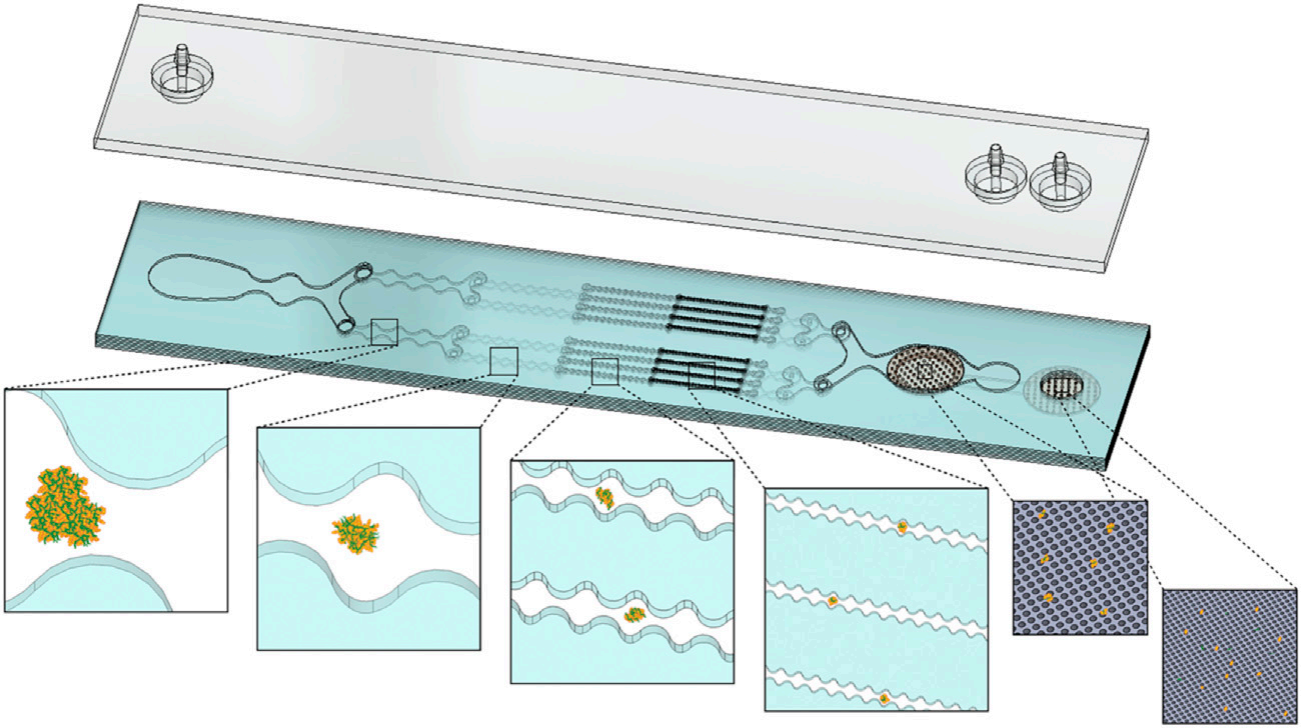
Schematic representation of the Integrated Disaggregation and Filtration (IDF) device. Large aggregates containing high extracellular matrix (ECM) content, such as digested tissue, are exposed to stepwise increases in shear stress throughout the branching channel array as the width narrows from 1 mm to 125 µm. Cell aggregates are held together via through cell-cell (dark orange perimeter) and cell-ECM (green fibers) interactions. As ECM is digested by collagenase, the channel array gradually reduces aggregate size via hydrodynamic shear forces. The smallest channels and nylon mesh membranes then break down the cell-cell interactions that hold together the smallest aggregates and clusters. Channels, cell aggregates, and membrane pore sizes are not shown to scale.

Figure 1 shows a schematic of the IDF device, as recently presented (Lombardo et al., 2021). This device combines the branching channel array and dual-filtration modules, which provides two distinct mechanisms for dissociation of cellular aggregates and/or digested tissue. The branching channels gradually and uniformly dissociate cellular aggregates via stepwise increases in shear stress as channels decrease in size from millimeters to hundreds of microns, and cross-sectional width is modulated to generate fluidic jets (Qiu et al., 2015; Qiu et al., 2018a). We anticipate that these shear stresses will release cells by surface erosion, without affecting those that are deeper within an aggregate. Filters have been integrated into microfluidic devices to provide high throughput cell manipulation for drug development studies and to reduce clogging (Schirhagl et al., 2011; Booth and Kim, 2012). Our filtration device utilizes two nylon mesh membranes for removal of large aggregates, as well as increasing cell yield via dissociation (Qiu et al., 2018b). We expect that cell release is caused by direct physical interaction with the nylon threads or related hydrodynamic effect. In either case, we anticipate that dissociation occurs for aggregates and clusters that are on the same size scale as the pores, which were 50 and 15 µm sizes for this device. The IDF was fabricated using a commercial laminate process, with channel features laser micro-machined into hard plastic layers that were aligned and bonded using pressure sensitive adhesive under pressure lamination. The goal of this study is to perform a detailed examination of the different dissociation mechanisms offered by the branching channel array and filtration modules, as a function of different operational conditions and cell aggregate types. This will allow us to explore the efficiency of these dissociation mechanisms in models with different levels of intercellular adhesion forces and extracellular matrix content.

### Optimization of the Branching Channel Array Using MCF-7 Cell Aggregates

**FIGURE 2:**
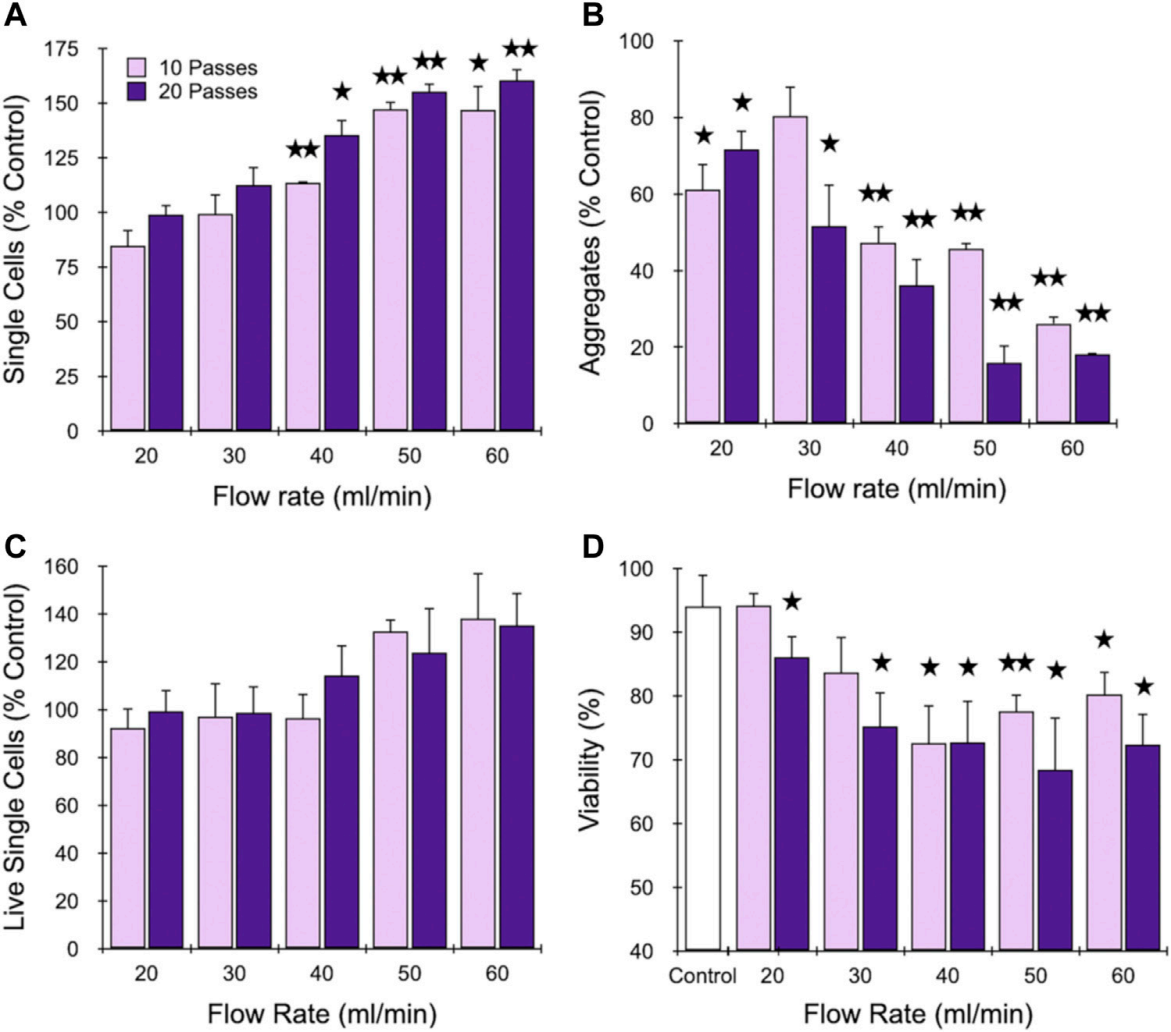
Optimization of the branching channel array using cell aggregates. MCF-7 cells were passed through the channels at different flow rates for either 10 or 20 passes. Results are shown for **(A)** total single cell, **(B)** aggregate, and **(C)** live single cell yields, which are all normalized to the control that did not pass through the device. Also presented is **(D)** single cell viability. Strong effects were observed for each metric above 40 ml/min, but higher pass number did not substantially influence results. Data are presented as mean values ± SEM from at least three independent experiments. Two-sided *t* test was used for statistical testing. Stars indicate *p* < .05 and double stars indicate *p* < .01 relative to the unprocessed control.

We began by investigating the branching channel array alone, which has been characterized in previous work using cell and tissue models, but under a limited set of operating conditions. Specifically, samples were either applied using a syringe pump at 10.5 ml/min flow rate and actuated back and forth through the device for up to 10 passes (Qiu et al., 2015; Qiu et al., 2018a) or using a peristaltic pump to recirculate at flow rates as high as 20 ml/min flow rate for up to 10 min (Lombardo et al., 2021). We chose to employ the syringe pump format because it can provide higher precision and control, and the branching channel array was isolated by using the first outlet, bypassing the filters (Figure 1). We used the MCF-7 breast cancer cell line because it provides a simple model with small cell aggregates and clusters that are comprised of minimal ECM (Carlo et al., 2006; Khademhosseini et al., 2006). This should provide insight into dissociation via the smaller channels and filters that result from disruption of cell-cell adhesions, which will help inform subsequent studies with tissue samples containing different cell types, higher ECM content, and overall greater complexity. MCF-7 cell suspensions were passed though the branching channel array using a syringe pump at flow rates ranging from 20 to 60 ml/min for either 10 or 20 passes, which is a far broader range than previously investigated. Single cell recovery was determined using a hemacytometer, and is presented in Figure 2A after normalization by the single cell count before device treatment. Single cells increased steadily with flow rate up to 50 ml/min before stabilizing at ∼150% of the control value, and all differences were statistically significant relative to the control at 40, 50, and 60 ml/min flow rates. Increasing device pass number tended to increase single cell number, but differences were modest and not statistically significant. Cell aggregates were also identified and quantified using the hemacytometer as containing 2 or more cells, and results are shown in Figure 2B, again after normalization by the control. As expected, aggregates decreased dramatically with flow rate, and in this case with pass number as well. Although not represented in the data, we also observed that most of the aggregates that remained after device processing were composed of only 2 or 3 cells, whereas the control had substantially larger aggregates of more than 10 cells (see Supplementary Material, Supplementary Figure S1). These findings demonstrate that the microchannel array reduced both aggregate number and size, corroborating the single cell data in Figure 2A for most conditions. However, we do note that the 20 and 30 ml/min conditions exhibited a decrease in aggregates that was statistically significant without generating more single cells. This may have been due to a secondary effect such as holdup within the branching channel array. Alternatively, cell reaggregation may have played a role. We generally assume reaggregation is unlikely since the buffer lacks divalent cations necessary for most cell-cell adhesion molecules, but a contributing factor could be DNA released from damaged cells that can cause cells to adhere together (Renner et al., 1993). We also note that reaggregation can be promoted under certain hydrodynamic conditions (Moreira et al., 1995). The channel constrictions and expansions provide elongational and shear flows similar to previous studies of colloidal aggregates (Harshe et al., 2011). Viable single cells were also identified using Trypan blue stain and counted (Figure 2C), and results were generally similar to total single cells in Figure 2A, but with smaller differences relative to the control. Finally, total viability was determined (Figure 2D), and we observed a decrease in viability with both flow rate and pass number, from the control value of ∼90% to as low as 70%. This shows that some damage can be associated with more aggressive dissociation conditions. Although these changes in viability are substantial, they were in line with our previous work using this MCF-7 aggregate model with the branching channel array under a recirculating flow format (Lombardo et al., 2021). This cell line model may be sensitive to mechanical dissociation due to the high intracellular adhesion forces, which can compromise the plasma membrane and allow the Trypan blue dye to enter the cell. Moreover, it is unclear whether this membrane effect is transitory, similar to shear flow-induced transfection methods (Stewart et al., 2018; Tay and Melosh, 2021; Aghaamoo et al., 2022).

### MCF-7 Aggregate Dissociation Using the Branching Channels and Filters

**FIGURE 3:**
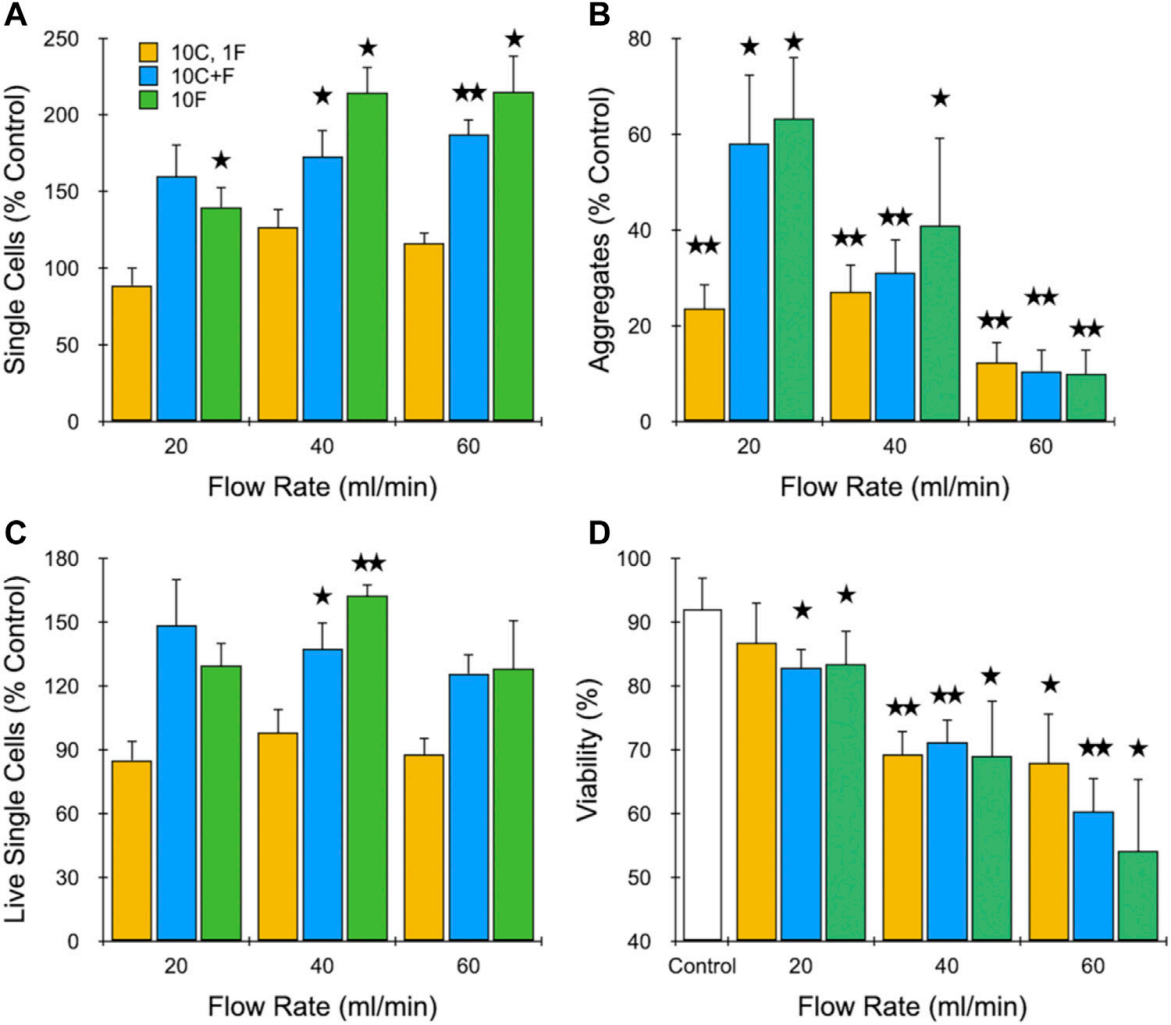
Evaluation of different dissociation formats using cell aggregates. MCF-7 cells were passed through the channel module 10 times and filter module once (10C, 1F), both channel and filter modules 10 times (10C + F), or the filter module 10 times (10F) at different flow rates. Results are shown for **(A)** total single cell, **(B)** aggregate, and **(C)** live single cell yeilds, which are all normalized to the unprocessed control. Also presented is **(D)** single cell viability. Optimal results were observed at 40 ml/min using multiple filter module passes. Data are presented as mean values ± SEM from at least three independent experiments. Two-sided *t* test was used for statistical testing. Stars indicate *p* < .05 and double stars indicate *p* < .01 relative to the unprocessed control.

Next, we evaluated the effect of filtration on dissociation of MCF-7 aggregates under different operational configurations, including with and without the branching channel array. Based on results from the previous section, we employed flow rates of 20, 40, and 60 ml/min. Moreover, we chose to limit pass number to 10, since 20 did not alter results substantially. Finally, we selected three different device configurations: 1) 10 passes through the channel array followed by 1 pass through the filters (10C, 1F), 2) 10 passes simultaneously through both the channel array and filters (10C + F), and 3) 10 passes through the filters alone (10F). Results obtained for single cells, aggregates, and viability are presented in Figure 3. Upon comparison of Figure 3A to Figure 2A, we found that a single filtration step offered no benefit to the branching channel array. In fact, the increase in single cell recovery that was observed at higher flow rates was now lost, which may have been due to greater sample holdup within the full IDF device. Passing sample through both devices in series enhanced performance, resulting in statistically significant increases in single cell recovery at 40 and 60 ml/min, with the latter approaching a 2-fold cell enhancement. Interestingly, single cell recovery was highest using the filtration module as a standalone treatment, exceeding 2-fold increases at both 40 and 60 ml/min. The presence of aggregates was primarily dependent on flow rate and not the device configuration (Figure 3B). However, aggregate values were lower when the branching channel array was used alone (see Figure 2B), particularly at the higher flow rates. We note that enhanced removal of aggregates by the filters may have been due to either dissociation or filtration effects. Viable single cell yield is shown in Figure 3C, and confirmed that the 40 ml/min flow rate with 10 filter pass conditions were optimal, as these were the only differences that were statistically significant relative to the control. Viability predominantly correlated with flow rate (Figure 3D), with the values at 40 ml/min exhibiting a similar ∼20% as in Figure 2C. At 60 ml/min, however, an additional decrease to ∼50% was observed for multiple filtration passes. Taken together, the surprisingly strong performance of filtration alone was likely heavily influenced by the MCF-7 model, with most aggregates starting at <100 µm and held together primarily by strong cell-cell adhesions. The filtration module clearly performed better on this sample type, which can likely be traced to the physical barrier mechanism provided by the sequential 50 and 15 µm mesh filters. The smallest feature size of the branching channel array is much larger at 125 μm, although shear stresses generated by the fluidic jets act on a smaller scale. Consequently, the channel dissociation mechanism does not add substantially to the filter dissociation mechanism with this cell aggregate model, and may have instead detracted from cell recovery via losses from device hold-up and/or damage. In sum, filtration is critical for dissociation of smaller aggregates and the 40 ml/min flow rate provides the optimal balance between promoting dissociation and limiting damage.

### Evaluation of Disaggregation and Filtration Using Digested Kidney Tissue

**FIGURE 4:**
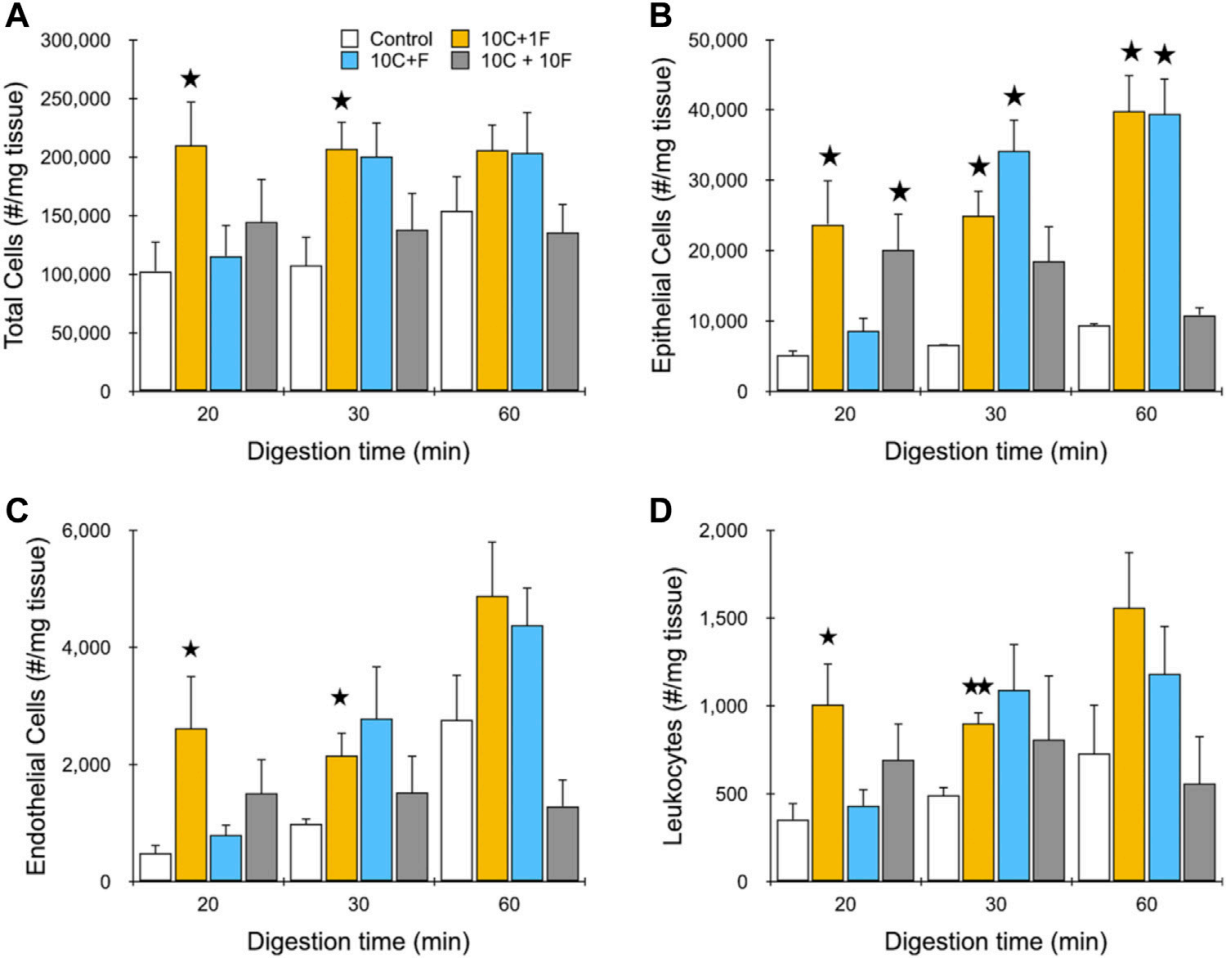
Evaluation of dissociation formats using murine kidney. Kidneys were harvested, minced, and digested for the indicated time periods. Samples were then passed through the channel module 10 times and filter module once (10C, 1F), simultaneously through both modules 10 times (10C + F), or sequentially through both modules 10 times (10C + 10F) at 40 ml/min and the resulting cell suspensions were analyzed using flow cytometry. Controls were pipetted/vortexed and passed through a cell strainer. Results are shown for viable and single **(A)** total cells, **(B)** EpCAM + epithelial cells, **(C)** endothelial cells, and **(D)** leukocytes. Optimal results were attained using the single filter pass at each digestion time. Data are presented as mean values ± SEM from at least three independent experiments. Two-sided *t* test was used for statistical testing. Stars indicate *p* < .05 and double stars indicate *p* < .01 relative to the control at the same digestion time.

Our next goal was to test the performance of the IDF device using murine kidney tissue that has been digested for different periods of time. Specifically, we chose to digest minced kidney with collagenase for 20, 30, or 60 min to produce aggregates of varying size and ECM content. Collagenase cleaves collagen, the major ECM structural element that provides tensile strength and anchors cells (Rozario and DeSimone, 2010; Zamprogno et al., 2021). Therefore, collagenase digestion weakens cell-ECM interactions, facilitating isolation of single cells via mechanical disaggregation, while maintaining the surface membrane and proteins intact (Reichard and Asosingh, 2019). Cell suspensions from digested tissue were loaded into the IDF device for processing at 40 ml/min flow rate under one of three configurations: 1) 10 passes through the channel array followed by 1 pass through filters (10C, 1F), 2) 10 passes simultaneously through both the channel array and filters (10C + F), and 3) 10 passes sequentially through the channel array and filters (10C, 10F). We chose not to test the filter device alone since digested tissue was likely to require the branching channel array to reduce larger fragments down in size before encountering the filters (see Figure 1), which would otherwise be retained on the mesh membranes via classical filtration. Flow cytometry was then used to identify and enumerate epithelial cells, endothelial cells, and leukocytes based on surface marker expression. Cell membrane proteins are vital for proper cell function and survival by mediating interactions with environmental cues and other cells (Liao et al., 2013), but can be affected by proteolytic digestion (Ohuchi et al., 1997), and therefore surface protein expression is an important functional metric. We also employed the viability stain 7-AAD. Live single cell counts are presented in Figure 4 for total cells and the 3 cell subtypes identified. For total cells (Figure 4A), we found that cell yield was highest using only a single filtration pass (10C, 1F). This result was surprising given the relatively poor performance of this condition with the MCF-7 model, but it was consistent across all digestion time points for kidney. At the 20 and 30 min digestion times, approximately 2-fold more single cells were recovered using the IDF device under the single filtration format relative to the respective controls (210,000 ± 30,000 verses 100,000 ± 20,000 per mg for 20 min digested sample; 210,000 ± 20,000 verses 110,000 ± 21,000 for 30 min digested sample), and differences were statistically significant. For the 60 min digestion time, single cells remained static, but the control increased to 150,000 ± 25,000 /mg, so the difference was not statistically significant. The simultaneous processing condition (10D + F) was similar to the control at the 20 min time point, but matched the single filtration case at both 30 and 60 min digestion times. The third and final condition, with separate and sequential 10 pass treatment through each module (10C, 10F), produced the lowest yield for each digestion time, which were all comparable to the corresponding controls. We conclude that these samples were over-processed, causing cell damage and lower overall yield. Epithelial cell results were very similar to total cells, but with substantially greater differences between the device conditions and controls (Figure 4B). This was likely due to the strong cell-cell adhesions holding epithelial cells together, which makes them more difficult to separate. Specifically, differences were in the range of 4- to 5-fold, and were statistically significant for both the single filtration (10C, 1F) and simultaneous processing (10C + F) formats at the 30 and 60 digestion times. However, only the single filtration (10C, 1F) format was statistically significant at the 20 min digestion time. We found that endothelial cell (Figure 4C) and leukocyte (Figure 4D) yields closely followed total cells. Viability data for each cell type is presented in the Supplementary Material, Supplementary Figure S2, and show that epithelial and endothelial cells maintained similarly high levels (>90%) for each IDF device processing format. Leukocyte viability was lower overall at ∼80%, but again was not affected by the IDF device. Taken together, these results suggest that shear forces from the branching channel array play a critical role in dissociation of minced and digested kidney tissue samples regardless of the degree of digestion, particularly for epithelial cells. Although not directly tested, we presume that the single pass through the filter device was beneficial, and at minimum ensured that a follow-up cell straining step was not necessary. Based on our data, passage through the filter component more than one time, either simultaneously or sequentially, would not be recommended for tissue due to limited benefit, or even detrimental effects, to cell yield and/or viability. We do acknowledge that this could change if less passes or lower flow rate was used with the branching channel array, now making multiple filter passes beneficial. Additionally, these results may have been influenced by the more abundant cell types, and there may be a smaller population of strongly cohesive cells that would benefit from multiple filter passes, which would require a higher resolution detection method such as single cell RNA sequencing.

### Optimization of Kidney Dissociation Using the IDF Device

**FIGURE 5:**
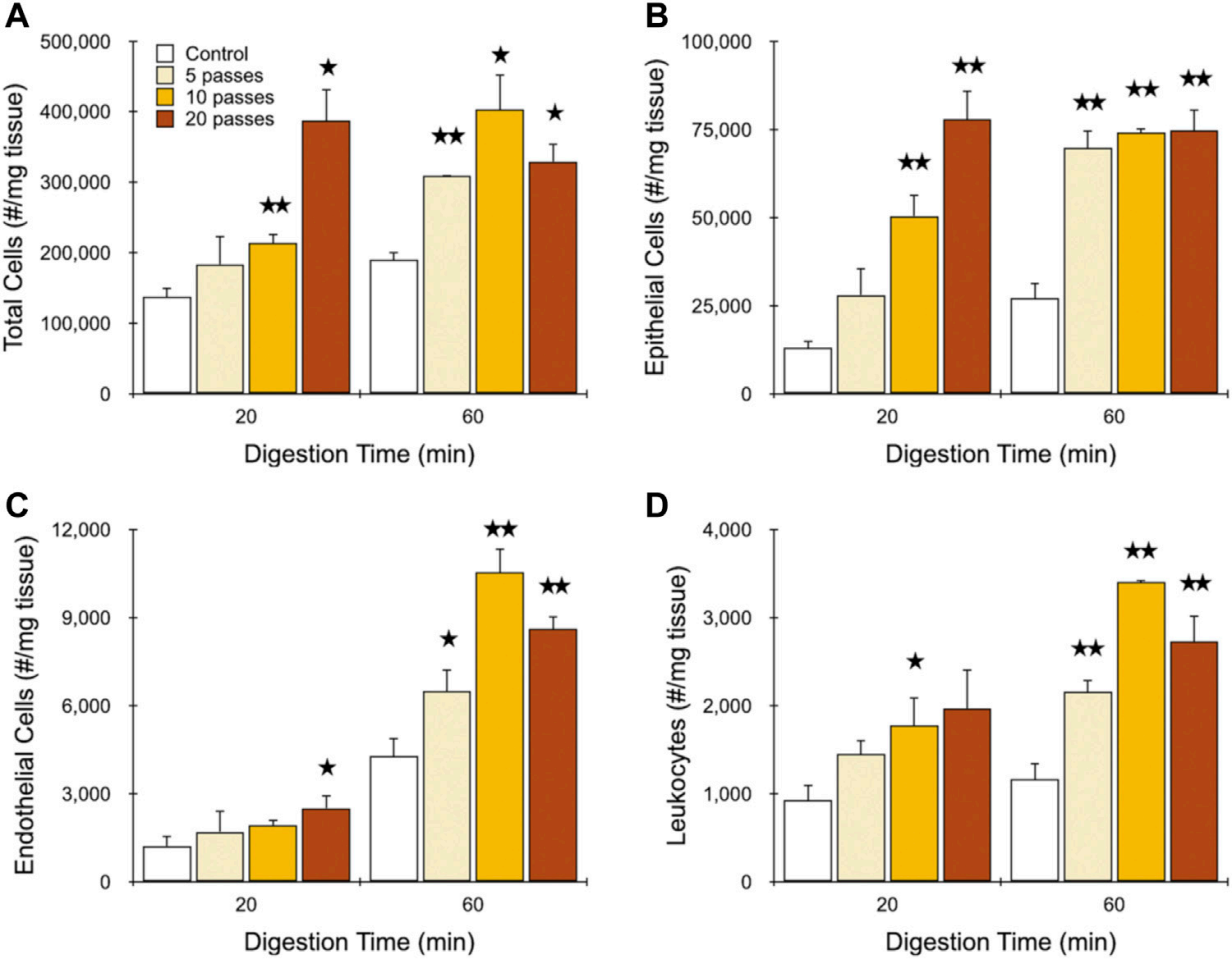
Final optimization of murine kidney. Kidneys were harvested, minced, and digested for the indicated time periods. Samples were then passed through the channel module for the indicated number of times and filter module once (10C, 1F) and resulting cell suspensions were analyzed using flow cytometry. Controls were pipetted/vortexed and passed through a cell strainer. Results are shown for viable and single **(A)** total cells, **(B)** EpCAM + epithelial cells, **(C)** endothelial cells, and **(D)** leukocytes. Similar epithelial yields were obtained after 20 min digestion time using 20 passes and 60 min digestion time using 10 passes. However, maximal endothelial and leukocyte yields required 60 min digestion time and 10 passes. Data are presented as mean values ± SEM from at least three independent experiments. Two-sided *t* test was used for statistical testing. Stars indicate *p* < .05 and double stars indicate *p* < .01 relative to the control at the same digestion time.

Finally, we sought to determine the most efficient operating conditions for the IDF device using minced and digested kidney. Based on the previous study, we expect that this would involve the branching channel array followed by a single pass through the filters. We again used different digestion times and a flow rate of 40 ml/min, but now varied branching channel array pass number from 5 to 20. We contend that this will provide the more controlled approach to modulate mechanical treatment level and subsequently identify optimal conditions for different tissue inputs and cell type outputs, as opposed to varying flow rate. Results for total cell recovery are presented in Figure 5A. After only 20 min of digestion, 5 and 10 passes through the branching channel array generated only modest increases in cell yield relative to the control, although the latter was statistically significant. However, increasing pass number to 20 enhanced cell recovery to nearly 3-fold that of the control. After a full 60 min digest, 5 passes produced a difference of 50% that was statistically significant. Further treatment initially increased cell yield to 2-fold before dropping back to ∼50% at 20 passes. Notably, cell recovery was the same for the 20 min digest using 20 passes as the 60 min digest with 10 passes, which would result in a substantial reduction in processing time without sacrificing performance. Findings were similar for epithelial cells (Figure 5B), but now with a more stepwise response observed for pass number after a 20 min digest, and no response at all after a 60 min digest. Importantly, equivalence in cell yield was preserved between the 20 min digest + 20 pass and 60 min digest + 10 pass conditions. We also note that the maximum epithelial cell recoveries of ∼75,000/mg tissue (78,000 ± 8,000/mg after 20 min digest and 20 passes; 75,000 ± 6,000/mg after 60 min digest and 10 passes) are substantially greater than the ∼40,000 to 60,000/mg tissue range from our previous works (Qiu et al., 2018a; Qiu et al., 2018b; Lombardo et al., 2021), highlighting the power of the IDF device when deployed in an optimal manner. Results for endothelial cells (Figure 5C) and leukocytes (Figure 5D) were comparable to total cells, however in both cases, additional mechanical processing could not compensate for shorter digestion time. The difference was particularly pronounced for endothelial cells, with ∼4-fold less cells obtained from the 20 min digest relative to the 60 min digest, even after 20 passes. Thus, the potential digestion time savings that the IDF device can offer to epithelial cells did not apply to all cell types. Viability data for each cell type is presented in the Supplementary Material, Supplementary Figure S3, and show that viability remained around 90% for total and epithelial cells under all conditions. A small decrease was observed for the 60 min digestion time for the IDF, which was statistically significant. Endothelial cells and leukocytes had substantially lower viability overall, but were also only weakly affected by IDF processing. These small changes in viability of a few percentage points was also observed in our previous work with the digestion device and, importantly, was not correlated with changes in stress responses by single cell RNA-sequencing (Lombardo et al., 2021). Although a consistent observation, the insensitivity of tissue cells to mechanical processing relative to the MCF-7 cell line is surprising. However, this may be influenced by the strong cell-cell adhesion strength and/or size (∼20 µm diameter) MCF-7 cells. Alternatively, this may simply reflect the different assay formats. Flow cytometry utilizes a gating scheme to remove cellular debris, which may very well contain the non-viable cells.

Tissues are composed of cells that are anchored to the ECM and/or neighboring cells via different types of adhesive interactions, including various integrins and cadherins, which includes complex structures such as focal adhesions, tight junctions, gap junctions, and adherens junctions (Yang and Weinberg, 2008; Gonzalez and Medici, 2014). Bearing this in mind, isolation of single cells from tissue can only ensue by overcoming all cell-cell and cell-ECM adhesions through chemical and/or mechanical means. Epithelial cells are typically arranged in sheets of cells that are connected to neighbors through cadherins and to the ECM through integrins. The adhesive force of epithelial cell-ECM interactions has been measured to be ∼250 nN, while epithelial cell-cell interactions were roughly similar at ∼100 nN (Maruthamuthu et al., 2011). Moreover, it has been shown that tissue digestion can result in heterogeneous distribution of ECM (Tseng et al., 2012). Hence, removing the cell-ECM interaction can provide an additive effect by making it easier to release epithelial cells from each other (Sander et al., 1998; Sakai et al., 2003; Shih and Yamada, 2012). We believe that these effects are represented in our data. After 60 min of digestion, collagen has largely been eliminated from the ECM, leaving only weakened cell-cell interactions that could be overcome by minimal mechanical processing with 5 passes through the branching channel array and 1 pass through the filter. Additional channel passes did not affect single cell yield, which may simply mean that cells were neither released nor damaged, although it is possible that these processes were in balance. This finding is most likely related to the fact that cell aggregates were smaller in nature after extensive digestion. Based on our results with MCF-7 cells, we would have expected that multiple passes through the filter device would have aided dissociation of smaller aggregates/clusters, but this was clearly not the case (Figure 4B). This may have been due to epithelial cells from kidney either being more sensitive to filter-induced damage or possessing weaker cell-cell adhesions that could sufficiently be overcome by the single filter pass. We acknowledge that this result may change for different tissues and/or cell types, such as tumors, which will be studied in future work. For the 20 min digestion time, substantial ECM still remained, and cell aggregates were presumably larger. This provided an opportunity for mechanical dissociation by the branching channel array to exert a key effect, releasing more single cells in a dose-dependent manner, culminating in the surprising result that 20 passes could rival the results after a full 60 min digestion. Shortening digestion time without compromising cell yield is of critical importance to limit the time that enzymes are in contact with cells, as well as stress response pathways that can interfere with transcriptomic analysis (Adam et al., 2017; van den Brink et al., 2017; O’Flanagan et al., 2019), as we have recently shown using the full microfluidic platform including the digestion device (Lombardo et al., 2021).

Endothelial cells and leukocytes displayed similar results, but with distinct differences to epithelial cells, as well as each other. These differences can likely be linked to their anatomic origin within tissues, namely within blood vessels, which are densely located throughout the kidney to facilitate the primary physiological role of blood filtration and waste removal. Importantly, blood vessels are a secondary structure within the tissue, requiring deeper access of enzymes and shear forces before chemical and/or mechanical dissociation can ensue. However, most leukocytes simply reside within the blood, which can be released as soon as that deeper access is attained. For these reasons, maximal leukocyte and endothelial recoveries after 20 min digestion could only reach ∼70 and ∼25%, respectively, of the 60 min digestion values. For endothelial cells, several molecules mediate cell-ECM interactions including proteoglycans and proteins, of which integrins are the best studied (Cines et al., 1998; Mehta and Malik, 2006). Interestingly, endothelial cells display an inherent shear stress sensitivity, including ECM remodeling (Jalali et al., 2001), stimulation of integrin-mediated cell-cell and cell-ECM adhesions (Moy et al., 2000), and relocation of adherins junction proteins (Chien et al., 2005; Hur et al., 2012). Although unclear at this time, a chemo-mechanical response may have played a role in the relatively poor performance of mechanical processing at short digestion times, when vessels were more intact. As stated, most CD45-positive leukocytes are present in free suspension within blood as monocytes, neutrophils, and lymphocytes. However, some can still be found throughout the tissue as resident tissue macrophages, T lymphocytes, or natural killer cells (Medzhitov, 2008; Park and Kupper, 2015), which would be anchored by cell-ECM interactions (E. Korpos et a., 2009; Masopust et al., 2010; Parker and Cox, 2020). It should be also considered that leukocytes can interact with endothelial cells through binding via junctional adhesion molecules at endothelial cell borders and surface receptors on leukocytes (Muller, 2011), which could cause some correlation.

Epithelial cells, endothelial cells, and leukocytes represented approximately one-quarter of the total cell count. The balance could include non-EpCAM expressing cells from the proximal tubules, distal convoluted tubules, loop of Henle, collecting duct, and mesangial cells, which comprise the bulk of cell subtypes detected using single cell RNA-sequencing by us and others (Rozenblatt-Rosen et al., 2017; Park et al., 2018; Liao et al., 2020; Lombardo et al., 2021). Additional structural cells such as podocytes, fibroblasts, and pericytes may have also contributed (Adam et al., 2017; Wu et al., 2019). Generally, these other cells displayed release dynamics that were most similar to EpCAM + epithelial cells.

## Conclusion

Herein, we have performed a detailed investigation of cell aggregate and tissue dissociation using the branching channel array and dual-filter modules that comprise the Integrated Disaggregation and Filtration device (IDF). We tested substantially higher flow rates than in previous work, and found that 40 ml/min was optimal for all samples. The dominant mechanism of dissociation varied, however, with smaller (<100 µm) and highly cohesive MCF-7 cell aggregates requiring multiple passes through the filters to achieve maximal single cell yield. Conversely, minced and digested murine kidney relied upon the branching channel array, and multiple filter passes was detrimental. This result was due to the larger size of the tissue aggregates and, as we hypothesize, a greater dependence on cell-ECM interactions. The most exciting finding was that the IDF device can release as many epithelial cells after a substantially shorter digestion period (i.e., 20 vs. 60 min) by simply passing through the device more times (i.e., 20 vs. 10). Reducing processing time in this manner could strongly impact long-term cell viability under culture settings and reduce stress responses that can interfere with transcriptomic-based cell classification, which will be studied in future work. Processing time with the IDF is otherwise similar to manual methods such as pipetting/vortexing and cell straining, on the order of a minute. Thus, the reduction in digestion time from 60 down to 20 min would represent a substantial savings. This result did not extend to endothelial cells, however, due to greater reliance on digestion, which could result in cell subtype biasing if this time-reduction strategy were employed. Overall, the optimal processing condition for all cell subtypes was to digest for 60 min, pass 10 times through the branching channel array at 40 ml/min, and then pass once through the filters at 40 ml/min. This work with the IDF device has enhanced our understanding of dissociation vis-à-vis different mechanisms and cell/tissue aggregate properties. In the future, we will continue to expand this knowledge by performing similar tests in conjunction with the digestion device, evaluating various tissues and enzyme solutions, and analyzing results using single cell RNA sequencing.

## Data Availability

The original contributions presented in the study are included in the article/Supplementary Material, further inquiries can be directed to the corresponding author.
