# Supplementary material for: Optimization of Mechanical Tissue Dissociation Using an Integrated Microfluidic Device for Improved Generation of Single Cells Following Digestion
Source: Front Bioeng Biotechnol. 2022 Feb 8;10:841046. doi: 10.3389/fbioe.2022.841046 (PMC8861371; doi:10.3389/fbioe.2022.841046)
Supplement: Supplementary file 1 [file DataSheet1.PDF]

Supplementary Material

Optimization of mechanical tissue dissociation using an integrated microfluidic device for improved generation of single cells following digestion.

Marzieh Aliaghaei[1] and Jered B. Haun[1,2,3,4,5*]

[1]Department of Chemical and Biomolecular Engineering, University of California Irvine, Irvine, CA, 92697, USA.

[2]Department of Biomedical Engineering, University of California Irvine, Irvine, CA, 92697, USA.

[3]Department of Materials Science and Engineering, University of California Irvine, Irvine, CA, 92697, USA.

[4]Center for Advanced Design and Manufacturing of Integrated Microfluidics, University of California, Irvine, Irvine, CA, 92697, USA.

[5]Chao Family Comprehensive Cancer Center, University of California, Irvine, Irvine, CA, 92697, USA.

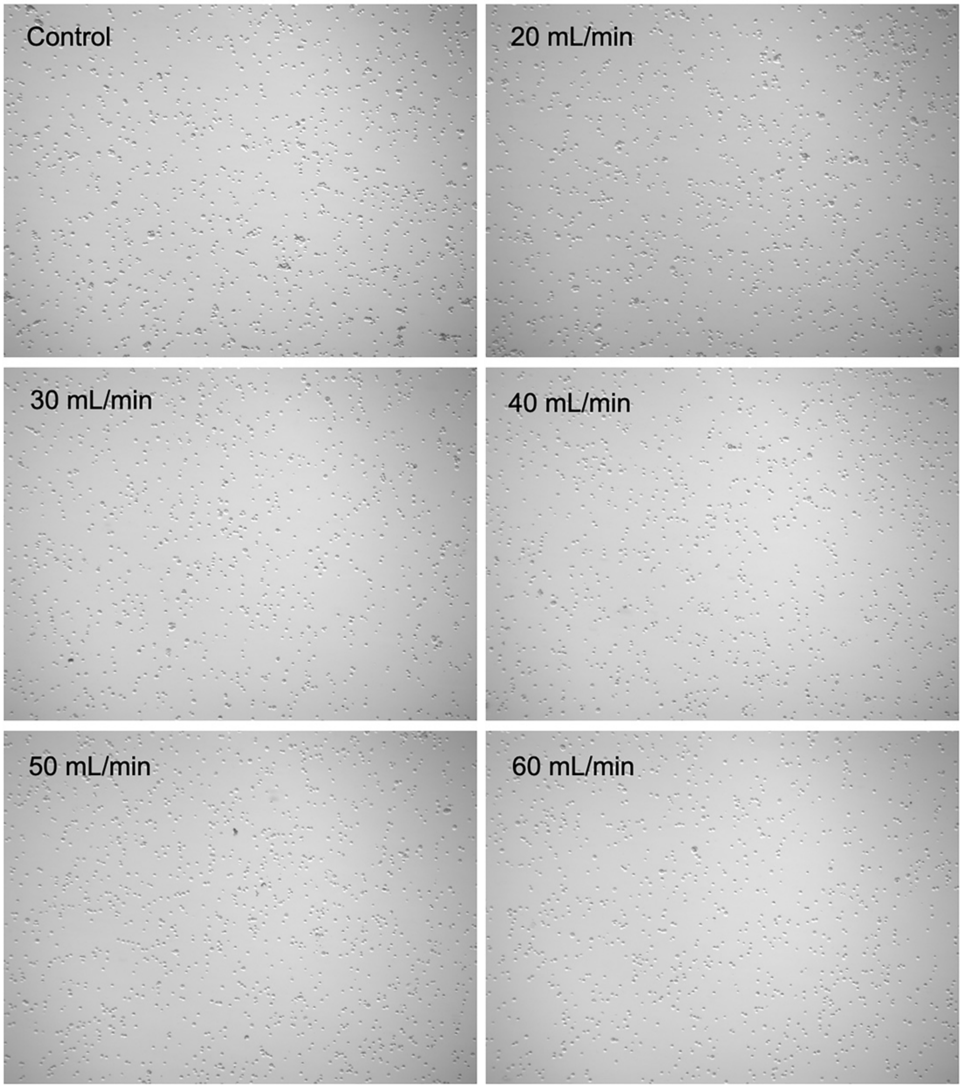

Figure S1. Micrographs of MCF-7 after processing with the branching channel array. MCF-7 cells were passed through the channels at the indicated flow rates for 20 passes. Single cell yield, aggregate yield, and viability are shown in Figure 2 of the main text. Many large aggregates can clearly be seen in the control, which decrease through the 20 and 30 mL/min flow rate conditions, and are rarely present above 40 mL/min flow rate. Results were similar after 10 passes.

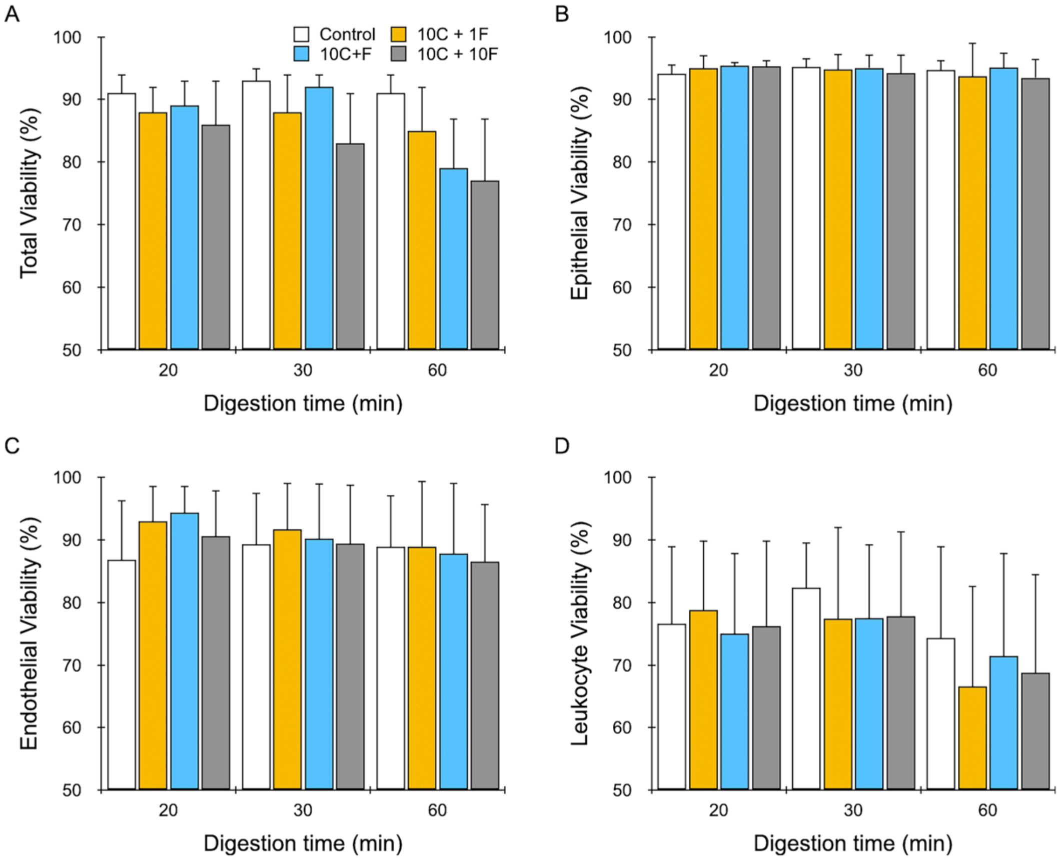

Figure S2. Cell viability from dissociation format study using murine kidney. Kidneys were harvested, minced, and digested for the indicated time periods. Samples were then passed through the channel module 10 times and filter module once (10C,1F), simultaneously through both modules 10 times (10C+F), or sequentially through both modules 10 times (10C+10F) at 40 ml/min and resulting cell suspensions were analyzed using flow cytometry. Controls were pipetted/vortexed and passed through a cell strainer. Cell viabilities are shown for (A) total cells, (B) EpCAM+ epithelial cells, (C) endothelial cells, and (D) leukocytes. Data are presented as mean values +/- SEM from at least three independent experiments. Two-sided T test was used for statistical testing, but no differences were statistically significant.

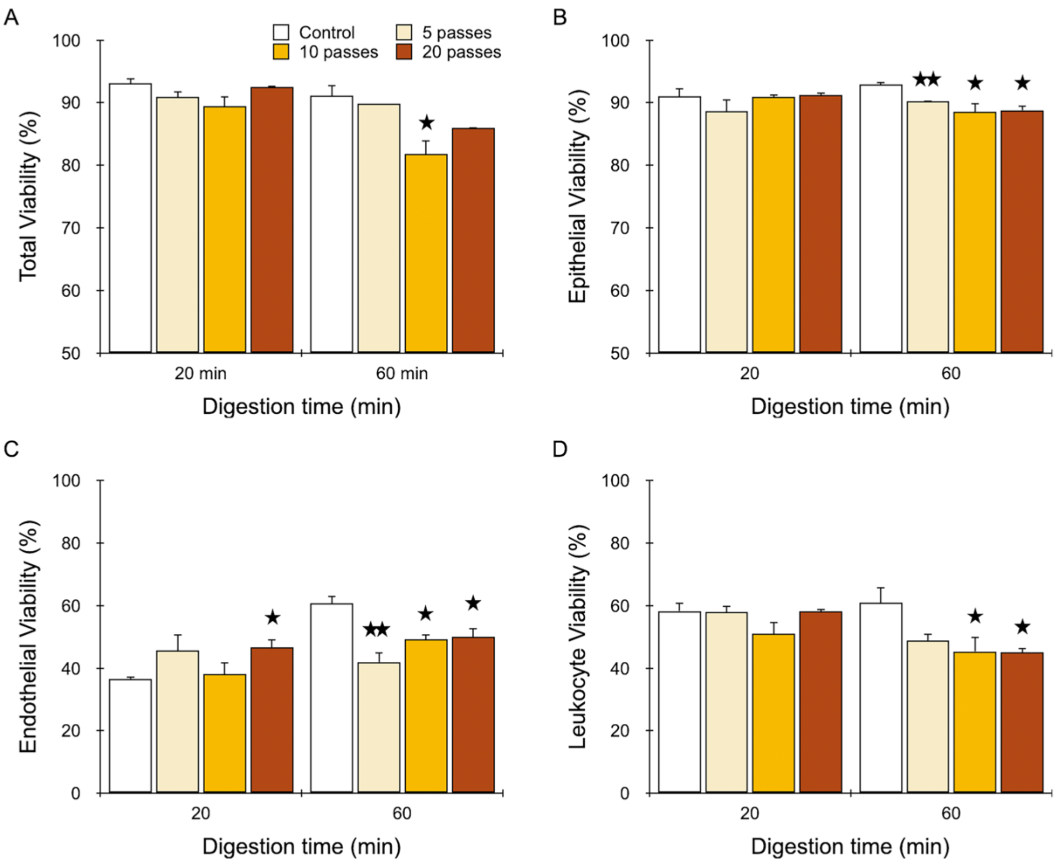

Figure S3. Cell viability from final optimization with murine kidney. Kidneys were harvested, minced, and digested for the indicated time periods. Samples were then passed through the channel module for the indicated number of times and filter module once (10C,1F) and resulting cell suspensions were analyzed using flow cytometry. Controls were pipetted/vortexed and passed through a cell strainer. Cell viabilities shown for (A) total cells, (B) EpCAM+ epithelial cells, (C) endothelial cells, and (D) leukocytes. Viabilities were generally lower after digesting for 60 min and using 10 device passes or more. Data are presented as mean values +/- SEM from at least three independent experiments. Two-sided T test was used for statistical testing. Stars indicate $p < 0.05$ and double stars indicate $p < 0.01$ relative to the control at the same digestion time.
